# A Novel SERRS Sandwich-Hybridization Assay to Detect Specific DNA Target

**DOI:** 10.1371/journal.pone.0017847

**Published:** 2011-05-31

**Authors:** Cécile Feuillie, Maxime Mohamad Merheb, Benjamin Gillet, Gilles Montagnac, Isabelle Daniel, Catherine Hänni

**Affiliations:** 1 Laboratoire de Géologie de Lyon - Terre Planètes Environnement, ENS Lyon, Université Lyon 1, CNRS, Ecole Normale Supérieure de Lyon, Lyon, France; 2 Institut de Génomique Fonctionnelle de Lyon, Université Lyon 1, CNRS, INRA, Ecole Normale Supérieure de Lyon, Lyon, France; 3 Plateforme nationale de Paléogénétique UMS PALGENE, CNRS - ENS de Lyon, Lyon, France; University of South Florida, United States of America

## Abstract

In this study, we have applied Surface Enhanced Resonance Raman Scattering (SERRS) technology to the specific detection of DNA. We present an innovative SERRS sandwich-hybridization assay that allows specific DNA detection without any enzymatic amplification, such as is the case with Polymerase Chain Reaction (PCR). In some substrates, such as ancient or processed remains, enzymatic amplification fails due to DNA alteration (degradation, chemical modification) or to the presence of inhibitors. Consequently, the development of a non-enzymatic method, allowing specific DNA detection, could avoid long, expensive and inconclusive amplification trials. Here, we report the proof of concept of a SERRS sandwich-hybridization assay that leads to the detection of a specific chamois DNA. This SERRS assay reveals its potential as a non-enzymatic alternative technology to DNA amplification methods (particularly the PCR method) with several applications for species detection. As the amount and type of damage highly depend on the preservation conditions, the present SERRS assay would enlarge the range of samples suitable for DNA analysis and ultimately would provide exciting new opportunities for the investigation of ancient DNA in the fields of evolutionary biology and molecular ecology, and of altered DNA in food frauds detection and forensics.

## Introduction

A wide variety of medical, diagnostic and industrial applications (detection of pathogens [1], [2], specific detection of mutations involved in human diseases [3], food quality control (GMO or allergen detection/quantification) [4] rely on nucleic acid analysis. In this context, molecular tools have flourished over the last 20 years [5], especially the development of the efficient and sensitive Polymerase Chain Reaction (PCR) [6], [7] procedure to detect minute amounts of DNA. PCR is used in several applied and fundamental research areas, such as paleogenetics. Practically, it consists in the authentication of a DNA sequence extracted from ancient remains (bones, teeth, coproliths) to solve important issues in evolutionary biology and molecular ecology, as DNA sequencing is one of the most efficient molecular methods for species identification. Indeed, species discrimination relies on the high nucleic variability of a specific gene. For instance, the gene encoding mitochondrial cytochrome c oxidase subunit 1 (COI) [8] is used in a specific PCR amplification of COI fragments combined with amplicon sequencing to identify species of the animal kingdom (DNA barcoding method [9]–[11]). Although highly efficient on well-preserved DNA templates, PCR often fails in amplifying ancient DNA molecules which are highly degraded and chemically modified since nucleic acids suffer a range of post-mortem degradations [12]–[17].

Indeed, two well-known types of DNA degradation, oxidized pyrimidines [13] and cross-links [12], can block the Taq Polymerase elongation activity. This suggests that use of an enzymatic amplification method (PCR, rolling circle [18]–[20], high-throughput sequencing [21], [22]) filters the DNA that is actually detected and studied and further that damaged DNA might be more widely distributed although unavailable for genetic analysis using current methods. Consequently, the development of a non-enzymatic method for detection of specific DNA, even highly degraded, could avoid long, expensive and inconclusive amplification trials. Furthermore, it would enlarge the range of remains suitable for analysis.

**Figure 1:**
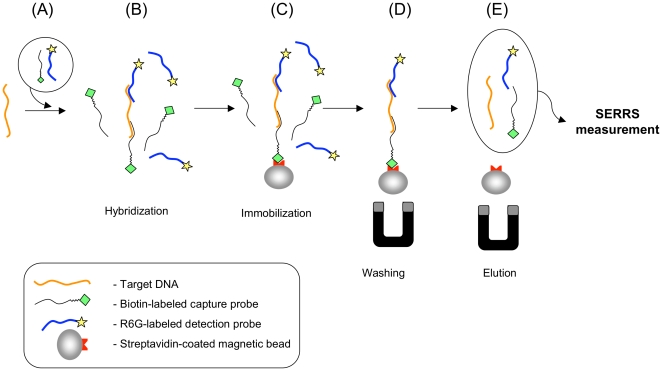
The sandwich-DNA hybridization assay principle. (A) Capture and detection probes are added to the solution of target DNA. (B) After the denaturation step (99°C, 10 minutes), the hybridization of the probes to the DNA target is achieved under gentle agitation (55°C, 3 hours). (C) Streptavidin-coated magnetic micro beads are added to the solution (gentle agitation for 30 minutes at room temperature). (D) Hybridized duplexes are captured by a magnet, unbound compounds are washed out. (E) Hybridized detection probes are eluted (95°C, 20 minutes) and are detected by SERRS in proportion of the initial concentration of target.

In this study, we have applied a Surface Enhanced Resonance Raman Scattering (SERRS) approach as an alternative technology to PCR amplification for the specific detection of DNA. SERRS is a vibrational spectroscopy technique whereby the Raman signal of the compound of interest can be amplified up to 10^14^ fold [23], [24]. SERRS-active molecules possess a chromophore with an absorption frequency close to the excitation frequency, and can adsorb on rough metallic surfaces such as colloidal silver nanoparticles. This adsorption has a doubly positive effect on the Raman signal: *(i)* it quenches the fluorescence that allows the highly specific Raman fingerprint of the molecule to be detected, *(ii)* it amplifies the Raman signal. Potential applications of SERRS detection have been under development since 1997 with a view of detecting DNA [25], thus becoming a rapidly emerging field [26]. Our present SERRS sandwich-hybridization assay is based on the specific hybridization of two nucleic probes to target DNA to be detected in solution (Figure 1). The nucleic probe labeled with rhodamine 6G (detection probe) allows the SERRS detection. The second probe, coupled with biotin (capture probe) allows immobilization and purification of the resulting hybridized complex (i.e. target DNA, capture and detection probes). Previous studies have demonstrated that SERRS-labeled synthetic DNA could be detected [25], [27], and that SERRS signal is stable after hybridization of a labeled oligonucleotide probe with a target DNA [26]. The sensitivity of SERRS makes it a valuable alternative non-enzymatic tool to detect DNA. We have developed a study model in which the target and control DNA are homologous sequences of chamois (*Rupicapra rupicapra*) and common goat (*Capra hircus*, a closely related species to *R. rupicapra*), respectively. The results of this new assay shows that DNA of *R. rupicapra* can be specifically discriminated from *C. hircus* at the 10^−8^ M level. Our SERRS sandwich-hybridization assay reveals its potential as a non-enzymatic alternative technology to DNA amplification methods (particularly the PCR method) for species detection with several application fields including: food frauds, forensics or studies of past populations through ancient DNA.

## Results and Discussion

### Principle of detection

We set up a procedure of double hybridization and immobilization of synthetic target DNA on magnetic microbeads (Figure 1). The capture probe, a biotin labeled sequence of 21 bases hybridizes with target DNA at the 5′ end, allowing its immobilization on the surface of the magnetic microbeads through the strong, non-covalent and thus reversible biotin-streptavidin interaction. The SERRS detection probe, a sequence of 22 bases labeled with a single rhodamine 6G (R6G) dye molecule hybridizes with target DNA at the 3′ end, allowing detection by Raman spectroscopy. After hybridization and fixation, the sandwich is separated using the magnetic microbeads and is washed to eliminate all unhybridized material. The sandwich is then subjected to denaturation to isolate magnetic microbeads, and to liberate the SERRS probe in solution. The supernatant containing the target DNA, and the complementary capture and detection probes, is then ready to be processed for SERRS measurements as described below.

### Concentration study and detection limit of the probe

**Figure 2:**
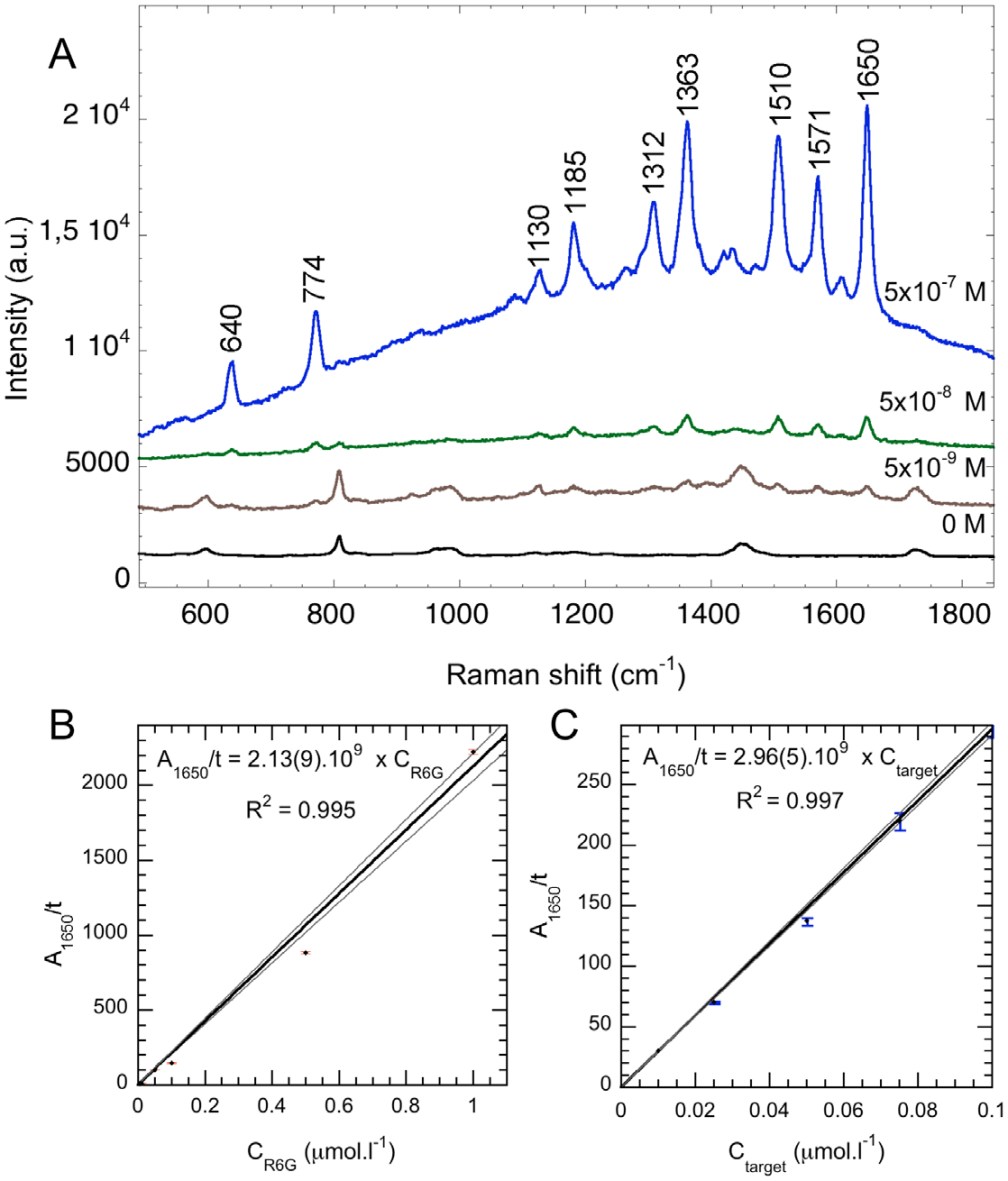
Specific SERRS signals. (A) SERRS spectra of the probe labeled with a single rhodamine 6G (R6G). The peak area beneath the most intense peak at 1650 cm^−1^ (*A*_1650_) is the parameter chosen for quantification. The black spectrum is the Raman signal of the PMMA cuvette, appearing in all spectra. Acquisition times for 0 M, 5×10^−9^ M, 5×10^−8^ M and 5×10^−7^ M are 1×15 s, 1×30 s, 1×10 s and 1×10 s, respectively. (B) Dilution study achieved on solutions of R6G probe. Calibration curve of *A*_1650_ normalized to the acquisition time in second, as a function of the initial concentration of the R6G probe (C_R6G_). Gray line represent the 1σ deviation. (C) Calibration curve of *A*_1650_ normalized to the acquisition time in second, as a function of the initial concentration of target DNA Gray lines represent the 1σ deviation.

A concentration study of the detection probe (*i.e.* nucleic probe labeled with the R6G dye) was performed for concentrations ranging from 10^−10^ to 10^−6^ M, without target DNA. The Raman fingerprint of the R6G probe and of the polymethyl methacrylate (PMMA) cuvette are displayed in Figure 2A. R6G is characterized by 9 Raman peaks, centered at 640, 774, 1130, 1185, 1312, 1363, 1510, 1571 and 1650 cm^−1^, respectively. Figure 2B plots the peak area beneath the most intense Raman band at 1650 cm^−1^, hereafter noted *A_1650_*, normalized to acquisition time *t*, as a function of the *C_R6G_* concentration of the detection probe. The ratio *A_1650_*/*t* varies linearly with the *C_R6G_* concentration, with a slope of 2.13(9)×10^9^ cts.s^−1^.M^−1^. This shows that the peak area beneath the most intense Raman band at 1650 cm^−1^ (*A*_1650_) allows quantification of the amount of DNA in the solution of interest.

The SERRS sandwich-hybridization assay developed in this study was undertaken on several samples of target *Rupicapra rupicapra* DNA of known concentrations, from 10^−6^ M to 10^−8^ M. Figure 2C plots *A*_1650_ normalized to acquisition time *t*, as a function of the *C_target_* concentration of the target DNA initially present in the sample. The *A*_1650_/*t* ratio varies linearly with the *C_target_* concentration, with a slope of 2.96(5)×10^9^ cts.s^−1^.M^−1^ indicating that this SERRS assay is not only a positive/negative test, but can also be used for quantitative purposes. The intensity of the SERRS signal obtained at the end of the protocol can be used to quantify the initial amount of target DNA.

The detection limit of the SERRS dye (*C_DL_*) was calculated from the spectrum acquired on the 10^−8^ diluted solution of detection probe as described below [28]:
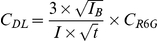
where *C_DL_* is the concentration at the detection limit, *I* the R6G dye signal intensity , *I_B_* the background intensity, *t* the acquisition time, and *C_R6G_* the initial concentration of R6G in the solution. We calculate a detection limit of 10^−10^ M, with *I* = 1768 cts, *I_B_* = 1442 cts, *t* = 30 s and *C* = 10^−8^ M, in agreement with the measured detection limit between 10^−11^ and 10^−10^ M.

### Specific detection and detection limit of *Rupicapra rupicapra* target

**Figure 3:**
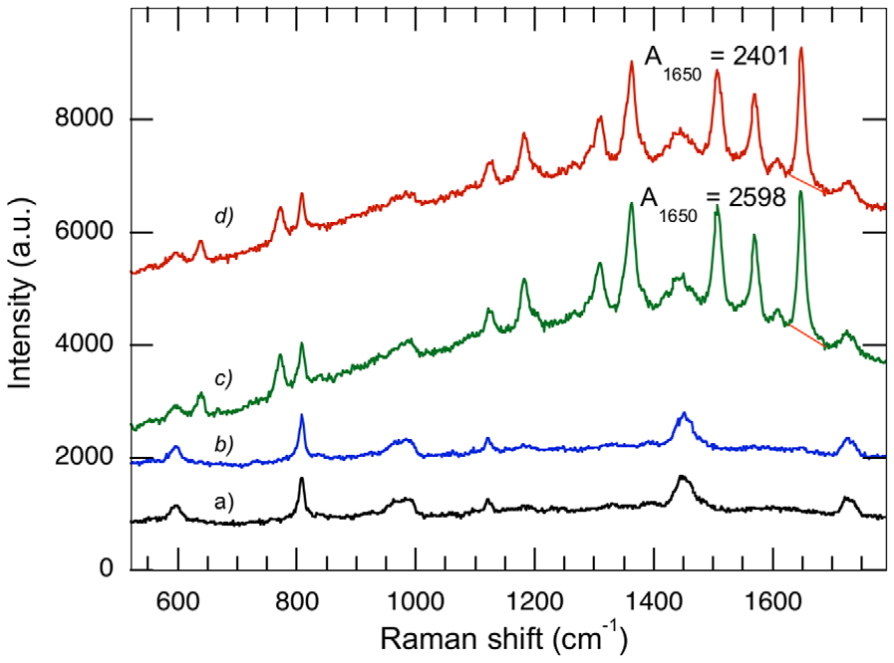
Specificity and co-contamination assays. All spectra were acquired in 1 acquisition of 30 s. (A) In black, negative control without target DNA. (B) In blue, *C. hircus*, negative control (5.10^−8^ M). (C) In green, signal from *R. rupicapra* (5.10^−8^ M). (D) In red, signal obtained from an equimolar mix of *R. rupicapra* and *C. hircus* DNA (5.10^−8^ M each).

We used the SERRS hybridization assay developed in the present study to detect DNA of chamois at concentrations ranging from 10^−6^ M to 10^−8^ M, respectively (Figure 2C and Figure 3). Negative controls were performed on solutions without target DNA, but containing both the capture and the detection probes, and then treated as described hereafter in experimental procedures (capture and wash steps). Measurements revealed only the plastic cuvette's signal and no specific Raman peak of R6G in the spectrum (Figure 3A). Figure 3C displays the Raman spectrum of the final elution of the SERRS sandwich-hybridization assay carried out on a 10^−8^ M DNA sample of *R. rupicapra*. An intense SERRS signal of the R6G dye is observed with some minor contribution of PMMA from the cuvette. The signal reveals the presence of detection probes that have been, first, hybridized to *R. rupicapra*, then, bound to the magnetic microbeads and finally, released in the elution supernatant. Thus, the SERRS signal indicates the presence of *R. rupicapra* DNA target in the sample.

We used the calibration curve from the concentration study (Figure 2C) to evaluate the yield of the present SERRS sandwich hybridization assay. The SERRS signal obtained after processing a sample of 10^−8^ M *R. rupicapra* leads to a calculated concentration of 1.15(2)×10^−8^ indicating that our assay allows for the detection of (*R. rupicapra*) DNA down to concentrations of 10^−8^ M with a yield approaching 100%. The calculated detection limit of our SERRS sandwich hybridization assay is 10^−10^ M of target DNA, which is in excellent agreement with the detection limit of the R6G SERRS probe, i.e. 10^−10^ M.

**Figure 4:**
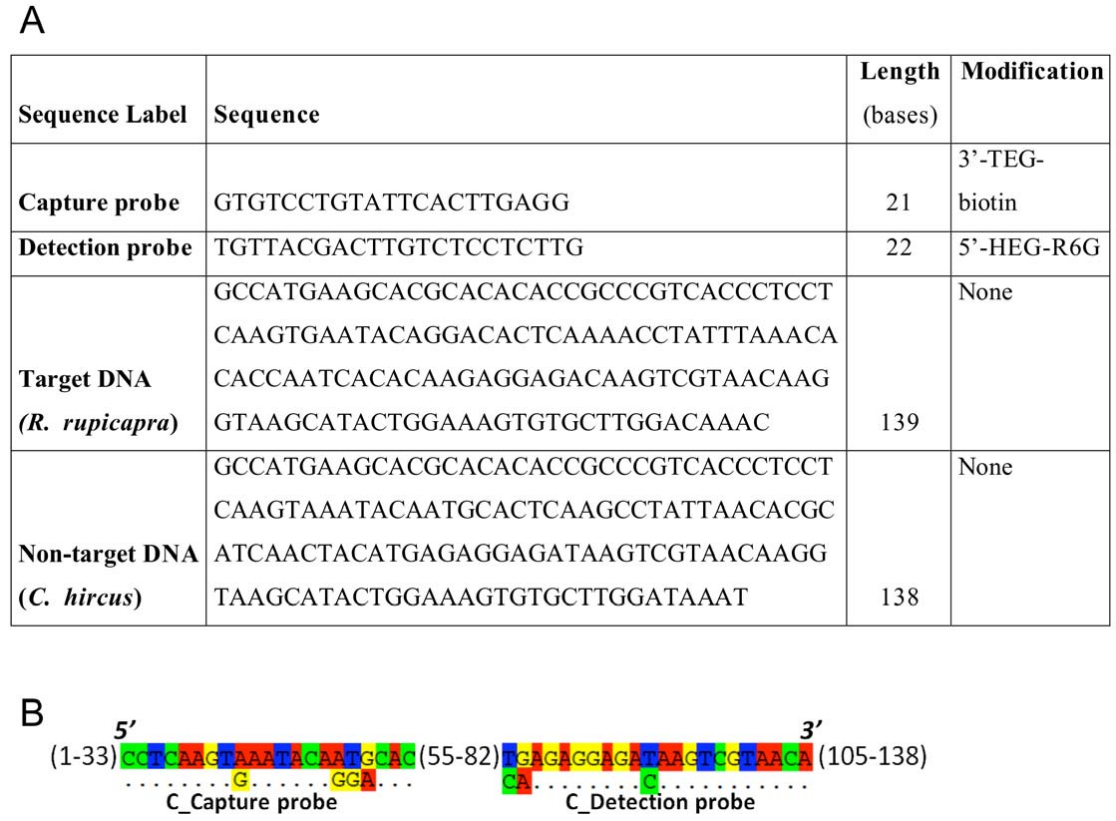
Probes and DNA characteristics. (A) Probes and DNA sequences. (B) Alignment of Non target DNA (*C. hircus*) sequence with complement sequences of Capture probe and Detection probe. Numbers present non-effective bases in the Non target DNA sequence.

To assess the specificity of the sandwich-hybridization assay presented in this study, we also tested the homologous region of mitochondrial DNA (12sRNA gene, 138 bp) of the domestic goat (*Capra hircus*) against chamois (*R. rupicapra*, 12sRNA gene, 139 bp) for which the SERRS detection was positive as presented above. More precisely, the sequence of *C. hircus* displays 4 mismatches with the capture probe, and 3 mismatches with the detection probe (Figure 4B). Samples containing either DNA of *C. hircus* only, or an equimolar mix of *R. rupicapra* and *C. hircus* DNA were investigated. The SERRS spectra obtained with 5×10^−8^ M *C. hircus* samples are similar to those of the negative control obtained without target DNA (Figure 3B). The absence of Raman signal from R6G clearly indicates that SERRS probes have been fully washed out during the elution process and confirms the specificity of the present sandwich hybridization assay (no unspecific/a-specific hybridization). Moreover, the Raman spectrum of the sample containing both *R. rupicapra* and *C. hircus* displays the same signal as the *R. rupicapra* sample only (Figure 3D and 3C respectively). This indicates that when the DNA (*C. hircus* in this study) present in a sample is not specific to the capture and detection probes (specific to *R. rupicapra* in this study), or when no DNA is present, the R6G SERRS signal is not observed in the elution solution. On the contrary, when the solution contains target DNA, with or without non-target DNA, a clear SERRS signal is observed (Figure 3), that allows quantification of the amount of target DNA.

Consequently, the sensitivity of the SERRS sandwich-hybridization assay we have developed allows the detection of single-stranded DNA with a yield close to 100%. This approach leads to specifically distinguishing DNA of very closely related species (*R. rupicapra* and *C. hircus*) at concentrations of at least 10^−8^ M.

The success of the present SERRS sandwich-hybridization assay in detecting specific DNA of *R. rupicapra* suggests that it could be developed and applied either as an initial test, prior to PCR, or as an alternative DNA detection method, when the activity of the Taq polymerase is inhibited [29], [30]. DNA extracts from many substrates (bones [31], teeth [32], coproliths [33], soil [34], sediments [35]…) are often co-extracted with PCR inhibitors especially in fields like paleogenetics [36]–[39], forensics [40] and medical analysis [41].

Such samples could be tested by the SERRS sandwich-hybridization assay to check the presence of specific target DNA, without a further DNA purification step, often critical for the recovery of DNA. For instance, environmental studies might benefit from this assay.

The most promising perspective for this SERRS sandwich-hybridization assay is that degraded DNA, unavailable for PCR amplification, could still be specifically detected. Indeed, the elongation by Taq polymerase is blocked if the template of interest contains consecutive abasic sites [42], cross-links [12], or a high amount of oxidized pyrimidines [13] – which is the case in most degraded substrates. This suggests that highly degraded DNA is currently only analyzed through the filter of PCR amplification whereas it may be widely distributed in substrates with levels of damage precluding genetic analysis by PCR. Our SERRS sandwich-hybridization assay, based on complementary hybridization, and therefore independent of the occurrence of abasic sites or oxidized bases, could allow for successful analysis of samples unsuitable for PCR, as it often occurs in paleogenetics. Samples that have been discarded from further PCR investigation because they appeared too degraded could be successfully analyzed by the SERRS sandwich-hybridization assay. As the amount and type of damage highly depends on the preservation conditions [16], the present SERRS assay would considerably enlarge the range of ancient samples suitable for DNA analysis.

## Materials and Methods

### Design of oligonucleotide probes

We used as target DNA a 139 base sequence of mitochondrial DNA (12sRNA gene) of *Rupicapra rupicapra* (chamois) (Figure 4A). Specificity controls were achieved by studying a homologous sequence of 138 bases of the same region of mitochondrial genome in *Capra hircus* (common goat). Chamois and goat were chosen because they are closely related (Figure 4B), and because none of them are usual contaminants (*e.g.* pig *Sus scrofa*, bovid *Bos taurus*, chicken *Gallus gallus*,…). These sequences as well as the SERRS 22-mer oligonucleotide labeled with one molecule of rhodamine 6G (detection probe), and the biotin labeled 21-mer oligonucleotide (capture probe) were purchased from Eurogentec®.

Sequences were aligned taking advantage of the Seaview software [43]. Capture and detection probes were designed to be specific of *R. rupicapra*. The 21-mer biotin probe is modified with 3′-biotin that is linked to the oligonucleotide via the 9-atom spacer: triethylene glycol (TEG) that provides the biotin with good accessibility to the streptavidin-coated magnetic microbeads. The 22-mer SERRS probe is modified with 5′-rhodamine 6G (R6G) which is linked to the oligonucleotide via the 18-atom spacer: hexaethylene glycol (HEG). Such a long spacer is necessary to avoid disturbing the hybridization when relatively high weight molecules like rhodamine 6G are linked.

### Reagents

All reagents were analytical grade. Sodium Dodecyl Sulfate 10% (SDS, #L4522), tetrahydrochloride spermine (Fluka, #85610), Polyoxyethylenesorbitan monolaurate (Tween 20, #P1379) and silver nitrate 99.999% (#S8157) were purchased from Sigma-Aldrich®. 1% trisodium citrate (#S1804) was from Fisher®. Ultra pure™ 20×SSC Buffer (Gibco, #15557-044), Streptavidin-coated magnetic microbeads (Dynal®, Dynabeads® MyOne™ Streptavidin C1, #650-02, 10 mg.ml^−1^, 2 ml, 7–12×10^9^ beads) and the matching DynaMag™-2 magnetic separator (Dynal®, #123-21D) were purchased from Invitrogen®.

### Sandwich hybridization and immobilization

A 4×SSC, Tween 20 (0.05%) buffer was used for hybridization, activation by washing the microbeads before use to remove the sodium azide from the storing buffer of the magnetic beads, immobilization and elution. The sandwich hybridization was performed in a single step as described hereafter: 10 µl of target DNA solution (concentration ranging from 10^−8^ to 10^−7^ M) were mixed with 10 µl of the capture probe and 10 µl of the detection probe (both in excess ×10, probes solutions concentration depending on the concentration of target DNA) in 30 µl buffer (Figure 1A) and heated to 99°C for 10 minutes to ensure denaturation of potential hairpins or autohybridized DNA. During the hybridization step, the samples were placed under continuous gentle stirring at 55°C for 3 hours (Figure 1B).

Meanwhile, series of 25 µl of the stock solution (10 mg/ml) of magnetic microbeads were rinsed three times in 25 µl of the buffer using the magnetic separator. Microbeads are then resuspended in 10 µl buffer, and added to the hybridization solution for immobilization under gentle continuous stirring for 30 minutes at room temperature (Figure 1C). The beads were finally washed twice in 200 µl of a 0.25×SSC, Tween 20 (0.5%) buffer using the magnetic separator to remove the unbound material (Figure 1D). The salinity of the washing buffer was chosen to optimize the specificity of our assay without any significant loss of material.

Prior to SERRS measurements, a final elution step is required to isolate the hybridized SERRS probes from the microbeads (Figure 1E). Beads re-suspended in 60 µl buffer are therefore heated at 95°C for 20 minutes for denaturation of both the DNA sandwich and the biotin-streptavidin bound [44]. The microbeads are then immobilized by the magnetic separator, and the supernatant is collected for SERRS measurements.

### SERRS measurements

The silver colloid used for SERRS measurements was prepared according to the Lee and Meisel protocol [45]. 90 mg of silver nitrate AgNO_3_ were dissolved in 500 ml of distilled water and subsequently heated under continuous stirring until ebullition. 10 ml of sodium citrate solution (1%) were then added. The solution was maintained at boiling for 90 minutes. The solution was clear at the beginning, and progressively took the appropriate grayish yellow color. The silver colloid has been stored in the dark at room temperature. The silver colloid aliquots used in this study were from the same batch.

The same protocol is used for measurements of detection probes in solution for both the R6G probe dilution assay and for the SERRS sandwich-hybridization assay. The eluted R6G probes contained in the supernatant were analyzed following a protocol modified from Faulds *et al.* [46]. Spermine concentration was decreased by one order of magnitude in order to minimize its contribution to the Raman signal. Nevertheless, it is still high enough *(i)* to act as an efficient aggregating agent of the silver colloid for generating hot spots and *(ii)* to neutralize DNA backbone negative charges allowing a better adsorption onto the metallic surface [25]. Thus, 20 µl of the supernatant was mixed with 20 µl of spermine (10^−2^ mol.l^−1^) in a single-use PMMA spectroscopy cuvette. 500 µl of silver colloid and 500 µl of distilled water were then added, and the solution was homogenized prior to SERRS measurement.

Samples were analyzed with a visible Jobin Yvon® LabRam HR 800 Raman spectrometer, coupled to a Spectra Physics® 2018Ar^+^/Kr^+^ laser tuned at 514.5 nm (LST, ENS de Lyon). The laser power on the sample was chosen between 1.5 and 2 mW. The spectra were acquired with a spectrometer grating of 600 gr.mm^−1^ centered at 1100 cm^−1^. Spectra were the result of 10×1 second to 1×30 seconds accumulation, depending on the concentration of the SERRS probe. Spectra were processed with Peakfit®, a software dedicated to peak detection, separation and analysis (Systat Software Inc).
